# Advancing Sustainable Aquaculture: Enhancing Production Methods, Innovating Feeds, Promoting Animal Welfare, and Minimizing Environmental Impact

**DOI:** 10.3390/ani15172601

**Published:** 2025-09-04

**Authors:** Cosmas Nathanailides

**Affiliations:** Department of Agriculture, University of Ioannina, 47100 Arta, Greece; nathan@uoi.gr

## 1. Introduction

Aquaculture has become the fastest-growing food production sector, driven by the rising global demand for seafood and the urgent need to reduce pressure on overexploited fish stocks. With global aquaculture production reaching an unprecedented 130.9 million tons in 2024, aquaculture is expected to further expand in the coming decade. Yet, this growth comes with challenges, including the reliance on fishmeal and fish oil, the impact of intensive farming on animal welfare, and the environmental costs of resource use and waste discharge. Addressing these challenges requires innovative approaches to nutrition, husbandry, and production systems that reduce ecological footprints while ensuring resilience and animal health.

The Special Issue “Advancing Sustainable Aquaculture: Enhancing Production Methods, Innovating Feed, Promoting Animal Welfare, and Minimizing Environmental Impact” was launched with this vision in mind. Its goal is to highlight new research that not only deepens scientific understanding but also delivers practical solutions for aquaculture sustainability. The nine articles published in this collection span key themes—feed innovation, stress physiology, and system-level integration—illustrating the breadth and dynamic nature of this rapidly developing field.

## 2. Contributions to the Special Issue

Feed formulation is at the heart of aquaculture sustainability. Several contributions examined novel ingredients and functional additives to reduce reliance on marine resources. Wang et al. [1] tested coconut oil as a fish oil replacement in grouper diets. Growth was unaffected, but high inclusion altered lipid metabolism and reduced long-chain PUFA in filets, highlighting trade-offs in nutritional quality. Gunathilaka et al. [2] supplemented seabream diets with gamma-aminobutyric acid (GABA) and sodium butyrate, showing enhanced growth, feed efficiency, and immune responses, thereby supporting soy protein concentrate as a viable fishmeal substitute. Jiang et al. [3] identified the optimal dietary lipids for mirror carp growth and antioxidant status while enhancing DHA/EPA deposition and avoiding excessive fat accumulation.

As aquaculture production intensifies, managing potential controlling factors or stressors such as temperature, oxygen, and light is crucial for welfare and productivity. Nie et al. [4] found that an 18 h photoperiod optimized survival, growth, immunity, and pigmentation in juvenile red-claw crayfish, offering practical guidance for crustacean culture. The study by Lahnsteiner [5] indicated that elevated temperature induced the structural and enzymatic remodeling of trout gills, demonstrating mechanisms of thermal adaptation but also revealing physiological limits under climate stress. Vega et al. [6] showed that hypoxia reduced the antibacterial activity of Chilean meagre mucus, though function recovered upon reoxygenation, highlighting both the resilience and vulnerability of mucosal immunity.

Innovative systems and improved early-life-stage practices are equally vital for sustainability. Janah et al. [7] examined mussel settlement success under different stocking densities, providing insights for efficient seed production in bivalve aquaculture.

Sommer et al. [8] demonstrated that nematodes can substitute for Artemia in shrimp nurseries reared under biofloc conditions, improving survival and offering a sustainable alternative to limited live feeds. Emerenciano et al. [9] compared aquaponics, hydroponics, and RAS with jade perch and lettuce. Mineral supplementation improved plant performance in aquaponics, while fish grew as well as in RAS, underscoring the promise of integrative food production.

The published studies presented in this Special Issue support the need for a multifaceted path to sustainability in aquaculture. Feed innovations demonstrate how alternative proteins, lipids, and functional additives can reduce dependence on fishmeal and fish oil while maintaining performance. Physiological studies provide tools to anticipate and manage stressors that will become more frequent under climate change and high-intensity farming. System-level innovations such as aquaponics and biofloc highlight how circular economy principles can improve efficiency, integrate plant and animal production, and reduce environmental impacts. At the same time, this Special Issue highlights ongoing needs, such as scaling up promising feed ingredients, ensuring long-term welfare outcomes across diverse species, and further minimizing ecological footprints. These directions call for continued interdisciplinary collaboration bridging nutrition, physiology, systems engineering, and environmental sciences.

I am pleased to present this Special Issue, which reflects the dedication and creativity of researchers worldwide committed to sustainable aquaculture. The nine papers published here demonstrate both the depth of scientific inquiry and the practical relevance of current innovation. I warmly thank the authors for their contributions, the reviewers for their rigorous evaluations, and the editorial team of Animals for their support throughout the process. I am also pleased to announce that a second edition of this Special Issue is now open for submission in *Animals*. I look forward to receiving new contributions that will continue to advance sustainable aquaculture practices, with a focus on nutrition, welfare, innovation, and environmental stewardship.
